# Validity of assistant technology for effective communication in emergency services

**DOI:** 10.1590/0034-7167-2024-0379

**Published:** 2026-01-26

**Authors:** Thayse Mota Alves, Betânia da Mata Ribeiro Gomes, Marisa Aparecida Amaro Malvestio, Simone Maria Muniz da Silva Bezerra, Josivan Soares Alves, Marina Gomes Costa, Débora Regina Alves Raposo, Cosme Michael Santos Farias

**Affiliations:** I Universidade de Pernambuco. Recife, Pernambuco, Brazil; II Universidade de São Paulo. São Paulo, São Paulo, Brazil; III Escola de Saúde Pública da Paraíba. João Pessoa, Paraíba, Brazil; IV Centro Universitário Unifacisa. Campina Grande, Paraíba, Brazil; V Universidade Federal de Campina Grande. Campina Grande, Paraíba, Brazil

**Keywords:** Nurses, Male, Patient Care, Emergency Medical Services, Emergency Nursing, Emergency Medical Service Communication Systems., Enfermeros, Atención al Paciente, Servicios Médicos de Urgencia, Enfermería de Urgencia, Sistemas de Comunicación entre Servicios de Urgencia.

## Abstract

**Objectives:**

to build and validate technology for communication among nurses in transition of care between pre-hospital services and emergency care through a committee of experts.

**Methods:**

a methodological study with a quantitative approach to develop and validate an assistive technology. The Delphi technique was used to reach consensus among experts, employing international sampling and snowball sampling. The analysis was performed using descriptive statistics and psychometric tests.

**Results:**

in the first round, 15 experts assessed the 138 items and suggested some adjustments. In the second round, a greater consensus was achieved, with a reliability index above 0.90 and a Cronbach’s alpha of 0.93, confirming the instrument’s validity.

**Final Considerations:**

the assistive technology developed showed potential for improving communication in transition of care in the emergency room, ensuring safety, continuity of care and offering a standardized interprofessional communication strategy.

## RESUMEN

**Objetivos:**

construir y validar tecnología para la comunicación entre enfermeras en la transición de atención entre servicios prehospitalarios y atención de emergencia, a través de un comité de expertos.

**Métodos:**

estudio metodológico con enfoque cuantitativo para desarrollar y validar una tecnología de asistencia. Se utilizó la técnica Delphi para alcanzar el consenso entre expertos, empleando muestreo internacional y muestreo por bola de nieve. El análisis se realizó mediante estadística descriptiva y pruebas psicométricas.

**Resultados:**

en la primera ronda, 15 expertos evaluaron los 138 ítems y sugirieron ajustes. En la segunda ronda, se logró un mayor consenso, con un índice de fiabilidad superior a 0,90 y un alfa de Cronbach de 0,93, lo que confirma la validez del instrumento.

**Consideraciones Finales:**

la tecnología de asistencia desarrollada mostró potencial para mejorar la comunicación en la transición de atención en urgencias, garantizando la seguridad, la continuidad de la atención y ofreciendo una estrategia de comunicación interprofesional estandarizada.

## INTRODUCTION

In 2004, the World Health Organization (WHO) established the World Alliance for Patient Safety, emphasizing the importance of risk-free healthcare and encouraging global action to mitigate and prevent avoidable harm in healthcare^(1,2)^. In line with this premise, Brazil created the National Patient Safety Program in 2013 to identify and monitor threats, improve care, and foster a culture of protection in healthcare facilities^(3)^.

In order to guarantee excellent care, the WHO defined six global patient safety objectives, with the main aspects being correct patient identification, effective communication among healthcare professionals, ensuring safety in prescribing and administering medications, practicing hand hygiene, and preventing falls and pressure injuries^(4,5)^.

Effective communication plays a crucial role in the healthcare sector and is a global priority, as it fosters interaction between patients and healthcare professionals, which is essential for effective and accurate care^(6)^. Effective communication helps reduce errors and increase patient safety^(7)^. Communication problems are among the main causes of incidents in the healthcare field, compromising quality of care^(2)^.

According to information from the Brazilian Health Regulatory Agency^(8)^, in 2018, it was found that 95.08% of adverse events in the healthcare sector occurred in hospitals and emergency rooms. Given this scenario, the Ministry of Health reformed the Brazilian National Emergency Care Policy and created the Emergency Care Network, aiming to improve and integrate access to emergency departments within the Brazilian Health System^(9)^.

Emergency departments handle complex and unpredictable cases, hindering interprofessional communication, especially during transitions between pre-hospital and in-hospital services^(10-12)^. High patient turnover and continuous interruptions in emergency departments compromise effective communication and continuity of care^(13)^.

Effective communication depends on technology, logistics, and protocols, but also on professionals dedicated to sharing patientand family-centered information^(14)^. Communication gaps during transitions of care are a leading cause of preventable deaths associated with adverse events^(15)^.

Effective communication-based articulations are crucial for coordination and continuity of care between different levels of care^(14)^. Studies highlight the importance of standardizing the care transition process among emergency departments^(16,17)^.

Manser^(18)^ highlights the importance of healthcare services research to improve the effectiveness and sustainability of interventions. The Country Cooperation Strategy for 2022-2027, aligned between the Brazilian Ministry of Health and PAHO/WHO, prioritizes research and development of health technologies^(19)^.

Nietsche *et al*.^(20)^ highlight the challenge of using and creating assistive technologies to improve nursing practice and interprofessional relationships. Without a defined protocol for this context, this research can support improvements in care transitions, developing assistive technology appropriate for emergency settings and ensuring patient safety.

Recognizing the lack of ideal, evidence-based solutions, it is necessary to develop an effective interprofessional communication strategy to address this complex problem, recognized as a priority by the WHO^(13,15,18)^. This research sought to contribute to the management and quality of care in emergency departments by adopting a standardized approach to reduce adverse events, improve patient safety, and increase professional satisfaction.

In this regard, the assistive technology developed in the present study was conceived in 2023 by a multidisciplinary team of researchers and healthcare professionals, aiming to optimize and standardize communication between nurses during transition of care in emergency care settings.

The instrument developed is structured around the organization and systematization of fundamental information, including patient identification, initial assessment, potential risks, and identification of professionals responsible for care. Its development followed a rigorous methodological process of review and validity by experts, ensuring its applicability and relevance for emergency clinical practice.

## OBJECTIVES

To assess the content validity and reliability index of assistive technology for effective communication among nurses during the transition of patient care between mobile prehospital services and other emergency departments, using a committee of experts.

## METHODS

### Ethical aspects

The project was submitted to the Research Ethics Committee of the *Hospital Universitário Oswaldo Cruz, Universidade de Pernambuco*, to ensure its compliance with ethical standards for research involving human subjects. The study was conducted in accordance with Resolution 510/2016 of the Brazilian National Health Council and began only after authorization from the REC-CONEP System. All participants provided electronic signatures on the Informed Consent Form (ICF). The data will be archived for at least five years, in accordance with Resolution 466/12 and REC/ENSP guidelines, after which it will be discarded.

### Study design, period and location

This is a methodological study of technological development, which involves the construction and validity of a healthcare assistive technology. According to Polit and Beck^(21)^, these studies mostly focus on the development of instruments, with a focus on processes of construction, validity, and assessment/application of tools, focusing on multiple target audiences^(22)^.

Nursing science has increasingly gained prominence nationally and internationally over the last decade, with a growing number of studies in the field of technology validity. According to Pasquali^(23)^, it is established that, when faced with a checklist, there are several methods for obtaining validity, such as construct validity, criterion validity, and content validity.

For this study, content validity was adopted, and the methodological proposal for this validity was conducted according to Pasquali’s psychometric framework^(23)^. In order to assess the instrument in the content validity dimension, Pasquali^(23)^ considers three fundamental criteria focusing on the characteristics of the population of interest, namely: language clarity, assessing the language used in items; practical relevance, considering the importance of each item in the instrument composition; and theoretical relevance, considering the degree of association between item and theory, focusing on the relationship between it and the construct.

The operationalization of this method was carried out using the Delphi technique, which is an investigative technique that allows the grouping of opinions of geographically dispersed experts, using careful procedures to generate a consensus on a given topic^(24)^. The aim was to obtain consensus on the instrument component items in a systematic manner, containing experts’ opinions in the field through successive rounds articulated via contact via exclusive email. At each round, a new link to Google Forms^®^ containing the updated validity form was sent.

### Population or sample; inclusion and exclusion criteria

Powell^(25)^ argues that, despite the lack of consensus on the ideal number of participants, emphasis should be on the panel quality, not necessarily on its statistical representativeness. Spínola^(26)^ and Faro^(27)^ add that there is no fixed number of experts, varying according to the phenomenon under study and selection criteria, with priority being given to the expertise, availability, and motivation of the experts involved in all stages of the study.

The research sample, which validated the new assistive technology, was intentionally composed. For recruitment, which took place in August 2023, and study systematization, two groups of experts emerged: group 1 (G1), composed of PhDs in the field, and group 2 (G2), composed of professionals working in the context of care practice.

Potential G1 members were selected through an active search on the Brazilian National Council for Scientific and Technological Development *Lattes* Platform, verifying the compatibility of the data found with the resumes available on the platform. Selection criteria included regarding the subject: development and validity of instruments in the healthcare field and/or patient care in care; regarding the level of education: PhD in the area; regarding nationality: Brazilian and foreign; regarding academic training/qualification: PhD; regarding professional activity: broad area - health sciences, area - nursing; regarding professional activity: all (teaching, research, healthcare practice); regarding the time for updating data: 12 months; regarding personal information: address, academic training/qualification, area of activity.

Initially, 146 professionals were identified. After reading and assessing the curriculum summaries contained in the platform (provided by the author or automatically generated by the *Lattes* Platform), those that did not present a description compatible with the research area of interest were excluded. Thus, a total of 20 potential G1 members were obtained.

These researchers were invited to participate in the study via email, accessed through the links in their respective resumes on the *Lattes* Platform. Professionals who did not return the email with the documents sent within the pre-established period of ten calendar days and/or who returned them without proper signature were excluded. The participation of experts working in the context of care practice in the care transition scenario is essential to ensure that the welfarist perspective of the service, faced in daily professional life, is also considered.

For G2, sampling was performed using the snowball sampling methodological technique, which, according to Polit and Beck^(21)^, is useful when seeking to assign specific similarities to participants within a group. Operationalization was done by nominating new members by the initial study members until the final number of 15 members was reached.

The experts who participated in the validity process were carefully selected to ensure that their practical experience, knowledge, and skills were relevant to the research topic. To begin recruitment, one professional from each state serving as the base for the UPE/UEPB Graduate Program in Nursing was intentionally selected. One professional was from the Mobile Emergency Care Service (In Portuguese, *Serviço de Atendimento Móvel de Urgência* - SAMU) in Paraíba and another from SAMU in Pernambuco (key informants), who nominated new professionals based on eligibility criteria.

Inclusion criteria for G2 participants were having worked as nurses at SAMU for at least one consecutive year and working in-person in transition of care to other emergency rooms. Exclusion criteria included professionals on vacation, leave, or away from work at the time of data collection. Official contact with these participants was maintained via email, provided during the study’s nomination.

With a total initial number of 35 invited experts, even anticipating abstentions during the rounds, as described by Scarparo *et al*.^(28)^, in which a reduction of 30% to 50% is expected in the first round and 20% to 30% in the second round, it would still be possible to reach double the minimum number in the end, according to the Pasquali model of psychometrics, which indicates the selection of six to 20 experts.

### Study protocol

Communication between the researcher and experts took place virtually, via a dedicated email address (transicaodecuidados@gmail.com). When participants responded to the invitation email regarding the first contact within the stipulated timeframe, they received a follow-up email containing an explanatory invitation letter and a link to Google Forms^®^. This free application allows for the management of surveys and online forms. The first version of the assistive technology was sent attached to the email, initiating the first round of validity.

By clicking on the Google Forms^®^ link, participants were redirected to a web page where they found a questionnaire consisting of four sections: ICF, where participants had to agree or disagree with the explicit content, this choice being essential for progression to the subsequent sections of the questionnaire. Data for characterizing participants included panelists’ sociodemographic, academic, and professional profiles. Guidelines for analyzing the instrument’s content, and the instrument itself were used by experts to validate the content of the domains and their items.

Data collection, guided by completing the online questionnaire, was operationalized by applying the Delphi technique^(27)^. To assess each domain, a Likert scale was used with degrees of consensus from 1 to 4, according to experts’ opinion on the item’s relevance, being: 1 - completely disagree; 2 - disagree; 3 - agree; 4 - completely agree.

According to Kayo and Securato^(29)^, the number of item validity rounds varies according to the nature of the group, its homogeneity, and the complexity of the topic. The deadline for completing the validity form was 15 calendar days, as recommended by Scarparo *et al*.^(28)^, as a sufficient period for participant feedback.

In the validity form return rounds, answers were tabulated, counted, and analyzed, resulting in the redefinition of items contained in the assistive technology. Questions that achieved consensus among participants were excluded from the assessment form and confirmed in the final version of the assistive technology.

### Analysis of results and statistics

The content validity of each item was analyzed by calculating the Content Validity Index (CVI) using a Likert-type scale, in which answers 3 and 4 indicate a high level of acceptance. According to Amaya^(30)^, an acceptable consensus index among experts should be at least 0.80, preferably above 0.90. Therefore, we adopted a minimum consensus value of 80% among experts.

The score calculation followed the method proposed by Polit and Beck^(21)^, adding the consensus of items marked “3” or “4” by experts and dividing by the total number of participating experts. The instrument’s reliability was assessed using Cronbach’s α test, which measures the reliability and internal consistency of measuring instruments, estimating reliability based on the correlation among answers using the same scale.

Based on the response options used (strongly disagree, disagree, agree, strongly agree), the adopted reliability values were >0.90 (excellent), >0.80 (good), and >0.70 (acceptable).

The data collected from experts were entered into a Microsoft Excel^®^ 2021 spreadsheet, and the results related to participant characteristics were analyzed using descriptive statistics. Categorical variables were expressed as frequencies and percentages, and numerical variables as means and standard deviations.

The Statistical Package for the Social Sciences version 20.0 was used to tabulate the data, applying statistical tests to obtain results presented in graphs, figures, and tables. This procedure aimed to verify relevant aspects of the research and facilitate understanding of the results obtained.

## RESULTS

### Experts

At this stage of the study, the selection of experts followed pre-established criteria in the methodology. Twenty invitation emails were sent to G1 and 15 to G2, totaling 35 invitations. Those who accepted received the Google Forms^®^ link for the first round of validity, along with the explanatory letter and the first version of the developed technology.

Three experts from G1 and 12 from G2 responded, totaling 15 professionals in the first round and 12 in the second. Experts’ ages ranged from 28 to 56, with an average of 37.5 years, and 60% were women. The majority (93.6%) reside in the Northeast region.

Academically, 60% of experts graduated from public institutions. All had at least five years of training, ranging up to 33 years. The majority held a *lato sensu* graduate degree (80%), followed by a master’s degree (40%), a PhD (20%), and a postdoctoral degree (6.7%).

Professionally, 93.3% work in nursing and/or teaching (46.7%), with 40% also in research and outreach. The majority work in the Northeast region, with 40% working in different locations.

The results of the first round of instrument validity are described below.

### Description of answers related to the first round of validity

In this phase, experts assessed 138 pieces of information, divided into nine stages: 1^st^ identification; 2^nd^ overview - occurrence kinematics; 3^rd^ overview - initial service (items “general condition”, “X”, “A”); 4^th^ overview - initial service (item “B”); 5^th^ overview - initial service (items “C”, “anatomical image with caption”); 6^th^ overview - initial service (item “D”); 7^th^ overview - initial service (item “E”); 8^th^ overview - initial service (item “F”); 9^th^ final (items “potential risks”, “professionals involved in the transition”).

Items were assessed for language clarity, practical relevance, and theoretical relevance using a 4-point Likert scale. After each stage, experts could suggest changes.

Table 1 shows the second stage (overview - occurrence kinematics) had an inadequate CVI for language clarity, requiring adjustments. The low standard deviation and high consistency in the answers indicated substantial consensus among experts on the remaining stages, which were considered clear and relevant.

**Table 1:** Description of answers related to the first round of validity

Stage	Items	Mean	SD	Answers				CVI
				1	2	3	4	
**1^st^ identification**	Language clarity	3.600	0.507	0	0	6	9	1.000
	Practical relevance	3.533	0.640	0	1	5	9	0.933
	Theoretical relevance	3.600	0.507	0	0	6	9	1.000
**2^nd^ overview - occurrence kinematics**	Language clarity	3.067	0.799	0	4	6	5	0.733
	Practical relevance	3.600	0.507	0	0	6	9	1.000
	Theoretical relevance	3.533	0.516	0	0	7	8	1.000
**3^rd^ overview - initial service (items “general condition”, “X”, “A”)**	Language clarity	3.600	0.507	0	0	6	9	1.000
	Practical relevance	3.600	0.507	0	0	6	9	1.000
	Theoretical relevance	3.667	0.488	0	0	5	10	1.000
**4^th^ overview - initial service (item “B”)**	Language clarity	3.733	0.458	0	0	4	11	1.000
	Practical relevance	3.600	0.507	0	0	6	9	1.000
	Theoretical relevance	3.733	0.458	0	0	4	11	1.000
**5^th^ initial service (items “C”, “anatomical image with caption”)**	Language clarity	3.533	0.640	0	1	5	9	0.933
	Practical relevance	3.600	0.507	0	0	6	9	1.000
	Theoretical relevance	3.667	0.488	0	0	5	10	1.000
**6^th^ overview - initial service (item “D”)**	Language clarity	3.400	0.828	0	3	3	9	0.800
	Practical relevance	3.533	0.640	0	1	5	9	0.933
	Theoretical relevance	3.600	0.632	0	1	4	10	0.933
**7^th^ overview - initial service (item “E”)**	Language clarity	3.667	0.617	0	1	3	11	0.933
	Practical relevance	3.733	0.458	0	0	4	11	1.000
	Theoretical relevance	3.733	0.458	0	0	4	11	1.000
**8^th^ overview - initial service (item “F”)**	Language clarity	3.333	0.724	0	2	6	7	0.867
	Practical relevance	3.533	0.640	0	1	5	9	0.933
	Theoretical relevance	3.600	0.632	0	1	4	10	0.933
**9^th^ final (items “potential risks”, “professionals involved in the transition”)**	Language clarity	3.733	0.594	0	1	2	12	0.933
	Practical relevance	3.800	0.414	0	0	3	12	1.000
	Theoretical relevance	3.867	0.352	0	0	2	13	1.000

*CVI - Content Validity Index; SD - standard deviation.*

Even with adequate CVI, some suggestions were accepted to improve practicality and safety, such as: 1^st^ stage: changing subitem “sex” to a marking option, including “mother’s name” and “occurrence identification number”; 5^th^ stage: changing symbols in the legend to better distinguish injuries; 6^th^ stage: complete description of less commonly used acronyms and reorganization of subitems for a logical sequence; 8^th^ stage: adjusting term “weight” to “approximate weight” in subitem “pediatric”; 9^th^ stage: inclusion of professional council in item “professionals involved in the transition”.

Moreover, formatting adjustments were made and subtitles were added to the end of the instrument.

### Description of answers related to the second round of validity

Due to inadequate CVI of the second stage (overview - occurrence kinematics), a new round of validity was carried out, incorporating experts’ suggestions: readjustment of term “kinematics” to “assessment”, since “kinematics” refers to the movement of bodies, suitable only for trauma mechanisms; inclusion of a subitem on the use of safety devices, to predict non-apparent injuries that can be caused by compression; addition of subitem “incidents with multiple victims” in the item type/reason for occurrence, due to the complexity and need to prioritize immediate interventions in such scenarios; emphasis on origin and destination in subitem “transportation” for greater clarity and complementarity of information; removal of subitem “private environment”, due to possible ambiguities with “healthcare service”, which may be private or occur in a private environment.

During the second round, 20% of experts abstained. The revised stage was submitted in a new online form, and experts were again able to suggest improvements. Table 2 shows an increase in the consensus index, with all items achieving CVI >0.9. There were no additional relevant suggestions, thus successfully validating all stages by experts.

**Table 2:** Description of answers related to the second round of validity

Stage	Items	Mean	SD	Answers				CVI
				1	2	3	4	
**2^nd^ overview - occurrence kinematics**	Language clarity	3.417	0.669	0	1	5	6	0.917
	Practical relevance	3.500	0.674	0	1	4	7	0.917
	Theoretical relevance	3.750	0.452	0	0	3	9	1.000

*CVI - Content Validity Index; SD - standard deviation.*

### Instrument reliability

In the instrument’s reliability analysis, measured by means of Cronbach’s alpha assessment, the result of 0.93 was obtained, indicating internal consistency and excellent instrument reliability.

Strengthening the reliability of the assessment performed, Cronbach’s alpha values were calculated if an item was removed (Table 3), indicating that values remain high, demonstrating that each item contributes significantly to the instrument’s internal consistency, being strongly correlated with each other and suggesting that they measure similar constructs, contributing consistently to the instrument’s overall validity.

**Table 3:** Cronbach’s alpha value according to item removal

Stage	Items	Cronbach’s alpha if item is removed
**Identification**	Language clarity	0.921
	Practical relevance	0.921
	Theoretical relevance	0.921
**Overview - occurrence kinematics**	Language clarity	0.922
	Practical relevance	0.919
	Theoretical relevance	0.920
**Overview - initial service (items “general condition”, “X”, “A”)**	Language clarity	0.924
	Practical relevance	0.922
	Theoretical relevance	0.922
**Overview - initial service (item “B”)**	Language clarity	0.922
	Practical relevance	0.922
	Theoretical relevance	0.924
**Overview - initial service (items “C”, “image”)**	Language clarity	0.921
	Practical relevance	0.919
	Theoretical relevance	0.919
**Overview - initial service (item “D”)**	Language clarity	0.923
	Practical relevance	0.922
	Theoretical relevance	0.920
**Overview - initial service (item “E”)**	Language clarity	0.918
	Practical relevance	0.917
	Theoretical relevance	0.917
**Overview - initial service (item “F”)**	Language clarity	0.924
	Practical relevance	0.919
	Theoretical relevance	0.919
**Final (items “potential risks”, “professionals involved in the transition”)**	Language clarity	0.924
	Practical relevance	0.920
	Theoretical relevance	0.920

## DISCUSSION

The complexity of task transitions directly impacts cognitive load, influencing the use of psychological resources such as memory, attention, perception, reasoning, and creativity during problem-solving^(31)^. The use of pre-printed sheets has been shown to be highly beneficial in improving information retention during transition, virtually eliminating data loss^(32)^. Checklists are emerging as an important resource in preventing adverse events and promoting patient safety, contributing to improved communication, incident prevention, and the generation of quality indicators, especially in emergency settings where care is complex and intense. Scientific evidence reinforces the importance of standardizing communication protocols among healthcare professionals^(33)^.

A literature review on transitional care reveals intercontinental concerns about existing gaps, highlighting the need for standardized communication tools for good healthcare practices. Concerns about care transitions were identified in various healthcare settings, including intensive and semi-intensive care, revealing the fragility of this topic at different levels of care complexity. A lack of systematization in information management can lead to communication failures, exposing patients to risks^(34)^.

The active participation of the entire healthcare team is crucial, with the identification of counterparts during handoff and the assumption of responsibilities to ensure continuity of care^(35)^. Mnemonics, while not primarily focused on care transitions, are useful for interprofessional communication, providing a structure for memorizing care stages. SBAR, for instance, is widely used and associated with improved patient safety^(36)^.

The construction of an effective instrument is based on the organization and grouping of information that facilitates critical thinking. Penedo^(37)^ states that, in nursing practice, systematization and scientific basis are essential for safe and targeted care. The instrument in this study, with four categories, such as identification, overview, potential risks, and responsible professionals, aims to standardize transfer of care among nursing professionals^(1)^.

Validity of new instruments is crucial and should be ensured through content validity^(21)^. In the Brazilian context, the nursing workforce is rejuvenating, with a female predominance, reflecting the historically female profession^(38,39)^. The inclusion of experts from different regions could enrich the instrument with diverse opinions and suggestions^(40)^.

Study participants’ educational diversity, with 94% having graduate degrees, reflects a wealth of experience and skills essential for scientifically grounded practice^(37,39)^. The simultaneous involvement of experts in care and teaching/research ensures theoretical judgment of the content and its practical applicability^(37)^. The concentration of participants in capital cities highlights the influence of working conditions and available resources^(40)^.

Refining a new instrument is challenging and requires validity to ensure that the content covers what it is intended to measure. The instrument was structured to mitigate the risk of incidents related to patient identification, communication failures, medication errors, incorrect surgical procedures, infections, and falls^(8)^.

The internal consistency assessment, with a Cronbach’s alpha coefficient of 0.93, reveals excellent internal consistency, ensuring valid and reliable results^(41)^. Experts’ suggestions were essential to improve subitems, resulting in pertinent and relevant content, successfully validated and aligned with scientific evidence.

### Study limitations

Limitations of this study include the subjectivity of the analysis, as experts’ assessments may be influenced by personal opinions, resulting in biases in the validity data. Furthermore, the limited number of experts (15 and 12) may not adequately represent the diversity of experiences, affecting the generalizability of the results. Content validity only addresses aspects of clarity, relevance, and relevance, without including other psychometric measures that could provide a more comprehensive assessment of effectiveness. Time and resource constraints may have limited the development and validity of the technology, impacting the depth of the study. Finally, implementing the technology in clinical practice may face challenges, such as resistance to change among professionals or lack of adequate training.

### Contributions to health, nursing or public policy

By providing assistive technology that synchronizes communication between nurses during care transitions, this study will contribute to nursing and reduce major errors and inefficiencies in the emergency room. Patient safety technology improves the quality and efficiency of care by providing clear and accurate information to reduce errors and promote a culture of safety. Furthermore, as an educational tool, it trains nurses in best practices for professional communication and collaboration and in managing the integration of healthcare services. As a nationally applicable tool, it can be used in diverse contexts and promote consistency and effectiveness.

## FINAL CONSIDERATIONS

Preventing errors and omissions in emergency departments requires a thorough understanding of their origins, which can include flawed thinking, slips, and lapses, exacerbated by factors such as fatigue, fast-paced workloads, overtime, work overload, and high staff turnover. Identifying a gap in patient safety during the transition of care between mobile prehospital services and emergency departments, it was deemed important to develop and validate an assistive technology that critically addresses key aspects of care delivery and ensures continuity of care at all levels.

“Assistive Technology for Effective Communication during Care Transitions” was created as a solution to improve the quality, effectiveness, and safety of care. This technology stands out as a low-cost and easy-to-apply strategy, promoting a culture of patient safety and strengthening interprofessional relationships.

The validity process revealed consensus among experts, with CVIs above 0.90 for the most part, reflecting its clarity, practical relevance, and theoretical relevance. Furthermore, the reliability analysis of the instrument, using Cronbach’s alpha, demonstrated a value of 0.93, demonstrating excellent internal consistency.

The results of this research demonstrate that the technology possesses content validity in terms of language clarity, practical relevance, and theoretical relevance, highlighting its importance. This technology stands out for offering a standardized interprofessional communication strategy, representing a valuable tool for national application that seeks to establish a secure communication process.

This instrument marks an important stage in the quest for systematized care that ensures comprehensive continuity of healthcare. In subsequent stages, the instrument will undergo further testing to assess its effectiveness when administered by emergency professionals.

However, it is important to recognize that all content validity studies can have limitations due to the subjective nature of expert analysis, which can result in biases. Therefore, while content validity provides information on the representativeness and clarity of each item based on expert input, it is still necessary to apply additional psychometric measures.

Therefore, to enable submission to other validity stages and future implementation in the routine of emergency professionals, it is recommended that new studies be carried out to seek further evidence of the instrument’s applicability.

## Data Availability

Not applicable.
