# Supplementary material for: Validity of assistant technology for effective communication in emergency services
Source: Rev Bras Enferm. 2026 Jan 26;79:e20240379. doi: 10.1590/0034-7167-2024-0379 (PMC12844540; doi:10.1590/0034-7167-2024-0379)
Supplement: Translation

# Translation: Validação de tecnologia assistencial para comunicação efetiva no serviço de emergência

**DOI:** 10.1590/0034-7167-2024-0379pt

**Authors:** Thayse Mota Alves, Betânia da Mata Ribeiro Gomes, Marisa Aparecida Amaro Malvestio, Simone Maria Muniz da Silva Bezerra, Josivan Soares Alves, Marina Gomes Costa, Débora Regina Alves Raposo, Cosme Michael Santos Farias

**Affiliations:** I Universidade de Pernambuco. Recife, Pernambuco, Brasil; II Universidade de São Paulo. São Paulo, São Paulo, Brasil; III Escola de Saúde Pública da Paraíba. João Pessoa, Paraíba, Brasil; IV Centro Universitário Unifacisa. Campina Grande, Paraíba, Brasil; V Universidade Federal de Campina Grande. Campina Grande, Paraíba, Brasil

**Keywords:** Enfermeiros, Assistência ao Paciente, Serviços Médicos de Emergência, Enfermagem em Emergência, Sistemas de Comunicação entre Serviços de Emergência.

## Abstract

**Objetivos:**

construir e validar tecnologia para comunicação entre enfermeiros na transição de cuidados entre o serviço pré-hospitalar e a urgência, mediante um comitê de especialistas.

**Métodos:**

estudo metodológico com abordagem quantitativa para desenvolver e validar uma tecnologia assistencial. Utilizou-se a técnica Delphi para alcance do consenso entre especialistas, empregando amostragem internacional e bola de neve. A análise foi realizada por estatística descritiva e testes psicométricos.

**Resultados:**

na primeira rodada, 15 especialistas avaliaram o total de 138 itens, sugerindo alguns ajustes. Na segunda rodada, ocorreu um maior consenso, com índice de confiabilidade superior a 0,90 e alfa de Cronbach de 0,93, confirmando a validade do instrumento.

**Considerações Finais:**

a tecnologia assistencial desenvolvida apresentou potencial para aprimoramento da comunicação na transição de cuidados na emergência, garantindo segurança, continuidade assistencial e oferecendo uma estratégia padronizada de comunicação interprofissional.

## INTRODUÇÃO

No ano de 2004, foi estabelecido pela Organização Mundial de Saúde (OMS) o *World Alliance for Patient Safety*, ressaltando a importância da assistência à saúde livre de riscos e incentivando ações globais para mitigar e prevenir danos evitáveis na área da saúde^(1,2)^. Em sintonia com essa premissa, o Brasil criou em 2013 o Programa Nacional de Segurança do Paciente, com o intuito de identificar e monitorar ameaças, aprimorar os cuidados, e fomentar uma cultura de proteção nas unidades de saúde^(3)^.

Com o intuito de garantir um atendimento de excelência, a OMS definiu seis objetivos globais de segurança do paciente, tendo como principais aspectos a correta identificação do paciente, a comunicação eficaz entre os profissionais de saúde, a garantia da segurança na prescrição e administração de medicamentos, a prática da higienização das mãos, e a prevenção de quedas e lesões por pressão^(4,5)^.

A comunicação eficiente desempenha um papel crucial no setor da saúde, sendo uma prioridade global, pois favorece a interação entre paciente e profissionais de saúde, fundamental para um cuidado eficaz e preciso^(6)^. A comunicação eficiente ajuda a reduzir falhas e a aumentar a segurança do paciente^(7)^. Problemas de comunicação estão entre os principais responsáveis por incidentes no campo da saúde, prejudicando a qualidade do atendimento^(2)^.

Segundo informações da Agência Nacional de Vigilância Sanitária^(8)^, no ano de 2018, foi constatado que 95,08% dos eventos adversos na área da saúde tiveram como cenário hospitais e locais de pronto atendimento. Diante desse cenário, o Ministério da Saúde promoveu uma reformulação na Política Nacional de Atenção às Urgências e criou a Rede de Atenção às Urgências, com o objetivo de aprimorar e integrar o acesso aos serviços de emergência e pronto atendimento no âmbito do Sistema Único de Saúde^(9)^.

Os serviços de urgência e emergência são responsáveis por casos complexos e imprevisíveis, dificultando a comunicação interprofissional, especialmente na transição de cuidados entre os serviços pré-hospitalares e intra-hospitalares^(10-12)^. A alta rotatividade de pacientes e as interrupções contínuas nas emergências comprometem a comunicação eficaz e a continuidade do cuidado^(13)^.

A comunicação eficiente depende da tecnologia, logística e protocolos, mas também de profissionais dedicados a compartilhar informações centradas no paciente e família^(14)^. Lacunas na comunicação durante a transição de cuidados são uma das principais causas de mortes evitáveis associadas a eventos adversos^(15)^.

Articulações baseadas em comunicação eficaz são cruciais para a coordenação e continuidade dos cuidados entre diferentes níveis de atenção^(14)^. Estudos ressaltam a importância de padronizar o processo de transição de cuidados entre serviços de emergência^(16,17)^.

Manser^(18)^ destaca a importância das pesquisas em serviços de saúde para melhorar a eficácia e sustentabilidade das intervenções. A Estratégia de Cooperação de Países para 2022-2027, alinhada entre o Ministério da Saúde do Brasil e a OPAS/OMS, prioriza a pesquisa e desenvolvimento de tecnologias em saúde^(19)^.

Nietsche *et al*.^(20)^ destacam o desafio de utilizar e criar tecnologias assistenciais para melhorar a prática profissional de enfermagem e as relações interprofissionais. Sem um protocolo definido para esse contexto, esta pesquisa pode fundamentar melhorias na transição de cuidados, desenvolvendo uma tecnologia assistencial adequada ao ambiente emergencial e garantindo a segurança do paciente.

Reconhecendo a falta de soluções ideais e baseadas em evidências, é necessário construir uma estratégia de comunicação interprofissional eficaz para abordar esse problema complexo, reconhecido como prioritário pela OMS^(13,15,18)^. Esta pesquisa buscou contribuir para a gestão e qualidade dos cuidados nas emergências, adotando uma abordagem padronizada para reduzir eventos adversos, melhorar a segurança do paciente e aumentar a satisfação profissional.

Neste sentido, a tecnologia assistencial desenvolvida no presente estudo foi concebida em 2023 por uma equipe multidisciplinar de pesquisadores e profissionais de saúde, visando otimizar e padronizar a comunicação entre enfermeiros durante a transição de cuidados em ambientes de urgência e emergência.

O instrumento desenvolvido estrutura-se em torno da organização e sistematização de informações fundamentais, incluindo identificação do paciente, avaliação inicial, potenciais riscos e identificação dos profissionais responsáveis pelo atendimento. Seu desenvolvimento seguiu um rigoroso processo metodológico de revisão e validação por especialistas, assegurando sua aplicabilidade e relevância para a prática clínica emergencial.

## OBJETIVOS

Avaliar a validação de conteúdo e o índice de confiabilidade de tecnologia assistencial para a comunicação efetiva entre enfermeiros durante a transição de cuidados do paciente entre os serviços de pré-hospitalar móvel e as demais portas de urgência e emergência, utilizando um comitê de especialistas.

## MÉTODOS

### Aspectos éticos

O projeto foi submetido ao Comitê de Ética em Pesquisa com Seres Humanos do Hospital Universitário Oswaldo Cruz da Universidade de Pernambuco, com a finalidade de garantir a sua adequação às normas éticas para pesquisa envolvendo seres humanos. O estudo foi conduzido de acordo com o disposto na Resolução nº 510/2016 do Conselho Nacional de Saúde, sendo iniciado somente após a autorização do Sistema CEP-CONEP. Todos os envolvidos deram sua assinatura eletrônica no Termo de Consentimento Livre e Esclarecido (TCLE). As informações serão mantidas arquivadas por pelo menos cinco anos, em conformidade com a Resolução nº 466/12 e diretrizes do CEP/ENSP, sendo posteriormente descartadas.

### Desenho, período e local do estudo

Trata-se de estudo metodológico de desenvolvimento tecnológico, que envolve a construção e validação de tecnologia assistencial em saúde. Segundo Polit e Beck^(21)^, esses estudos se concentram, em sua maioria, no desenvolvimento de instrumentos, com foco nos processos de construção, validação e avaliação/aplicação de ferramentas e produtos com foco em múltiplos públicos-alvo.^(22)^

A ciência da enfermagem tem se destacado no cenário nacional e internacional de forma crescente na última década, com progressivo número de trabalhos no campo da validação de tecnologias. De acordo com Pasquali^(23)^, é estabelecido que, diante de um *checklist*, existem alguns métodos para a obtenção da validação, como a validade de construto, a validade de critério e a validade de conteúdo.

Para este estudo, foi adotada a validação de conteúdo, e a proposta metodológica para essa validação foi conduzida de acordo com o referencial psicométrico de Pasquali^(23)^. Destinando-se à avaliação do instrumento na dimensão da validação do conteúdo, Pasquali^(23)^ considera três critérios fundamentais com foco nas características da população de interesse, a saber: clareza de linguagem, avaliando a linguagem utilizada nos itens; pertinência prática, ponderando sobre a importância de cada item na composição do instrumento; e relevância teórica, ao considerar o grau de associação entre o item e a teoria, com foco na relação entre ele e o construto.

A operacionalização desse método foi realizada utilizando a técnica de Delphi, que se trata de técnica investigativa que permite o agrupamento de opiniões de especialistas dispersos geograficamente, utilizando-se procedimentos criteriosos para gerar um consenso sobre determinada temática^(24)^. Vislumbrando a obtenção do consenso sobre os itens componentes do instrumento, de forma sistematizada, contendo o parecer de especialistas na área através de sucessivas rodadas articuladas via contato através do e-mail exclusivo. A cada rodada, foi encaminhado um novo *link* do aplicativo *Google Forms* ^®^ contendo o formulário validativo atualizado.

### População ou amostra; critérios de inclusão e exclusão

Powell^(25)^ argumenta que, apesar da falta de consenso sobre o número ideal de participantes, a ênfase deve ser na qualidade do painel, não necessariamente em sua representatividade estatística. Spínola^(26)^ e Faro^(27)^ acrescentam que não existe um número fixo de especialistas, variando conforme o fenômeno em estudo e os critérios de seleção, sendo a prioridade atribuída à *expertise*, disponibilidade e motivação dos especialistas envolvidos em todas as etapas do estudo.

A amostra da pesquisa, que realizou a validação da nova tecnologia assistencial, foi composta de maneira intencional. Para o recrutamento, realizado em agosto de 2023, e a sistematização do estudo, foram constituídos dois grupos de especialistas: grupo 1 (G1), composto por doutores na área, e grupo 2 (G2), composto por profissionais que atuam no contexto da prática assistencial.

A inclusão dos potenciais integrantes do G1 foi realizada por meio de busca ativa na Plataforma *Lattes*, do Conselho Nacional de Desenvolvimento Científico e Tecnológico, verificando a compatibilidade dos dados encontrados com os currículos disponíveis na plataforma. Os critérios de seleção incluíram quanto ao assunto: construção e validação de instrumentos na área da saúde e/ou assistência ao paciente na urgência e emergência; quanto ao nível de formação: doutores na área; quanto à nacionalidade: brasileira e estrangeira; quanto à formação acadêmica/titulação: doutorado; quanto à atuação profissional: grande área - ciências da saúde, área - enfermagem; quanto à atividade profissional: todas (ensino, pesquisa, prática assistencial); quanto ao tempo de atualização dos dados: 12 meses; quanto às informações pessoais: endereço, formação acadêmica/titulação, área de atuação.

Inicialmente, foram identificados 146 profissionais. Após a leitura e avaliação dos resumos curriculares contidos na plataforma (informados pelo autor ou gerados automaticamente pela Plataforma *Lattes*), foram excluídos aqueles que não apresentavam descrição compatível com a área de interesse da pesquisa. Dessa forma, obteve-se um total de 20 potenciais integrantes do G1.

Esses pesquisadores foram convidados via e-mail, acessado nos *links* de seus respectivos currículos na Plataforma *Lattes*, para participar da pesquisa. Foram excluídos os profissionais que não retornaram o e-mail com os documentos enviados no prazo preestabelecido de dez dias corridos e/ou que os devolveram sem a devida assinatura. A participação de especialistas que atuam no contexto da prática assistencial no cenário da transição de cuidados é fundamental para que a perspectiva assistencialista do serviço, enfrentada no cotidiano profissional, também seja contemplada.

Para o G2, a amostragem foi realizada através da técnica metodológica de amostragem bola de neve (*snowball*) que, segundo Polit e Beck^(21)^, é útil quando se busca atribuir aos participantes semelhanças específicas a um grupo. A operacionalização foi feita mediante a indicação de novos membros pelos membros iniciais do estudo até o alcance do número final de 15 integrantes.

Houve uma seleção detalhada dos especialistas que participaram do processo de validação, garantindo que sua experiência prática, conhecimento e habilidade estivessem relacionados ao tema da pesquisa. Para iniciar o recrutamento, foi eleito intencionalmente um profissional de cada estado base do Programa de Pós-Graduação em Enfermagem UPE/UEPB, sendo um profissional do Serviço de Atendimento Móvel de Urgência (SAMU) Paraíba e outro do SAMU Pernambuco (informantes-chave), que indicaram novos profissionais conforme os critérios de elegibilidade.

Os critérios de inclusão para os participantes do G2 foram trabalhar como enfermeiros no SAMU há no mínimo um ano consecutivo e atuar presencialmente na transição de cuidados para as demais portas de urgência e emergência. Os critérios de exclusão incluíram profissionais em período de férias, licença ou afastados do serviço no momento da coleta dos dados. O contato oficial com esses participantes foi mantido via e-mail, fornecido durante a indicação para participação na pesquisa.

Com um número inicial total de 35 especialistas convidados, mesmo prevendo abstenções durante as rodadas, conforme descrito por Scarparo *et al.* ^(28)^, em que se espera uma redução de 30% a 50% na primeira rodada e de 20% a 30% na segunda rodada, ainda seria possível ao final atingir o dobro do número mínimo, conforme o modelo de Pasquali da psicometria, que indica a seleção de seis a 20 especialistas.

### Protocolo do estudo

A comunicação entre a pesquisadora e os especialistas ocorreu virtualmente, por meio de um correio eletrônico exclusivo para esse fim (transicaodecuidados@gmail.com). Ao responderem ao e-mail convite referente ao primeiro contato dentro do prazo estipulado, os participantes recebiam um retorno com outro e-mail contendo uma carta-convite explicativa e o *link* para um formulário do *Google Forms^®^.* Este aplicativo gratuito permite o gerenciamento de pesquisas e formulários de preenchimento *online*. Anexado ao e-mail, foi enviada a primeira versão da tecnologia assistencial elaborada, iniciando a primeira rodada de validação.

Ao clicar no *link* do *Google Forms^®^*, o participante era redirecionado para uma página da *web,* onde encontrava um questionário composto por quatro seções: TCLE, onde o participante tinha que concordar ou discordar do conteúdo explicitado, sendo essa escolha indispensável para a progressão nas seções subsequentes do questionário. Dados para caracterização dos participantes abrangeram perfil sociodemográfico, acadêmico e profissional dos painelistas. Orientações para análise do conteúdo do instrumento e o instrumento foram utilizados pelos especialistas para validação de conteúdo dos domínios e seus itens.

A coleta de dados, guiada pelo preenchimento do questionário *online*, foi operacionalizada pela aplicação da técnica Delphi^(27)^. Para a avaliação de cada domínio, foi utilizada a escala Likert com graus de consenso de 1 a 4, conforme a opinião dos especialistas sobre a relevância do item, sendo: 1 - discordo totalmente; 2 - discordo; 3 - concordo; 4 - concordo totalmente.

Segundo Kayo e Securato^(29)^, o número de rodadas de validação dos itens varia de acordo com a natureza do grupo, sua homogeneidade e a complexidade do assunto. O prazo para preenchimento do formulário validativo foi de 15 dias corridos, conforme recomendado por Scarparo *et al*.^(28)^, como um período suficiente para o *feedback* dos participantes.

Nas rodadas de devolução do formulário validativo, as respostas foram tabuladas, contabilizadas e analisadas, ocorrendo a redefinição dos itens contidos na tecnologia assistencial. As questões que obtiveram consenso dos participantes foram excluídas do formulário avaliativo e confirmadas na versão final da tecnologia assistencial.

### Análise dos resultados e estatística

A validade de conteúdo de cada item foi analisada por meio do cálculo do Índice de Validade de Conteúdo (IVC), utilizando uma escala do tipo Likert, em que as respostas 3 e 4 indicam um alto índice de aceitação. Segundo Amaya^(30)^, um índice de consenso aceitável entre os especialistas deve ser, no mínimo, de 0,80, preferencialmente acima de 0,90. Portanto, adotamos um valor mínimo de consenso de 80% entre os especialistas.

O cálculo do escore seguiu o método proposto por Polit e Beck^(21)^, somando os consensos dos itens marcados com “3” ou “4” pelos especialistas e dividindo pelo número total de especialistas participantes. A confiabilidade da ferramenta foi avaliada pelo teste α de Cronbach, que mede a confiabilidade e consistência interna de instrumentos de medição, estimando a confiabilidade com base na correlação entre as respostas que utilizam a mesma escala.

Com base nas opções de resposta utilizadas (discordo totalmente, discordo, concordo, concordo totalmente), os valores de confiabilidade adotados foram >0,90 (excelente), >0,80 (boa) e >0,70 (aceitável).

Os dados coletados dos especialistas foram inseridos em uma planilha eletrônica do *Microsoft Excel* ^®^ 2021, e os resultados relativos à caracterização dos participantes foram analisados por meio de estatística descritiva. As variáveis categóricas foram expressas em frequências e porcentagens, e as variáveis numéricas em médias e desvios padrão.

Para a tabulação dos dados, utilizou-se o *Statistical Package for the Social Sciences*, versão 20.0, aplicando-se testes estatísticos para obter resultados apresentados em gráficos, figuras e tabelas. Esse procedimento visou verificar aspectos relevantes da pesquisa e facilitar a compreensão dos resultados obtidos.

## RESULTADOS

### Especialistas

Nesta etapa do estudo, a seleção dos especialistas seguiu critérios pré-estabelecidos na metodologia. Foram enviados 20 e-mails de convite para o G1 e 15 para o G2, totalizando 35 convites. Os que aceitaram receberam o *link* do *Google Forms* ^®^ para a primeira rodada de validação, junto com a carta explicativa e a primeira versão da tecnologia elaborada.

Responderam três especialistas do G1 e 12 do G2, totalizando 15 profissionais na primeira rodada e 12 na segunda. A faixa etária dos especialistas variou de 28 a 56 anos, com média de 37,5 anos, sendo 60% mulheres. A maioria (93,6%) reside na região Nordeste.

Em termos acadêmicos, 60% dos especialistas se formaram em instituições públicas. Todos tinham no mínimo cinco anos de formação, variando até 33 anos. A maioria possuía pós-graduação *lato sensu* (80%), seguida por mestrado (40%), doutorado (20%) e pós-doutorado (6,7%).

Profissionalmente, 93,3% atuam na enfermagem assistencialista e/ou docência (46,7%), com 40% também na pesquisa e extensão. A maioria atua na região Nordeste, com 40% trabalhando em diferentes localidades.

A seguir, são descritos os resultados da primeira rodada de validação do instrumento.

### Descrição das respostas relativas à primeira rodada de validação

Nesta fase, os especialistas avaliaram 138 informações, divididas em nove etapas: 1ª identificação; 2ª visão geral - cinemática da ocorrência; 3ª visão geral - atendimento inicial (itens “estado geral”, “X”, “A”); 4ª visão geral - atendimento inicial (item “B”); 5ª visão geral - atendimento inicial (itens “C”, “imagem anatômica com legenda”); 6ª visão geral - atendimento inicial (item “D”); 7ª visão geral - atendimento inicial (item “E”); 8ª visão geral - atendimento inicial (item “F”); 9ª final (itens “potenciais riscos”, “profissionais envolvidos na transição”).

Os itens foram avaliados quanto à clareza de linguagem, pertinência prática e relevância teórica, usando uma escala Likert de 4 pontos. Após cada etapa, os especialistas podiam sugerir alterações.

Na Tabela 1, a 2ª etapa (visão geral - cinemática da ocorrência) teve um IVC inadequado para clareza de linguagem, exigindo ajustes. O baixo desvio padrão e a alta consistência nas respostas indicaram consenso substancial entre os especialistas nas demais etapas, que foram consideradas claras e relevantes.

**Tabela 1:** Descrição das respostas relativas à primeira rodada de validação

Etapa	Itens	Média	DP	Respostas				IVC
				1	2	3	4	
**1ª identificação**	Clareza de linguagem	3,600	0,507	0	0	6	9	1,000
	Pertinência prática	3,533	0,640	0	1	5	9	0,933
	Relevância teórica	3,600	0,507	0	0	6	9	1,000
**2ª visão geral - cinemática da ocorrência**	Clareza de linguagem	3,067	0,799	0	4	6	5	0,733
	Pertinência prática	3,600	0,507	0	0	6	9	1,000
	Relevância teórica	3,533	0,516	0	0	7	8	1,000
**3ª visão geral - atendimento inicial (itens “estado geral”, “X”, “A”)**	Clareza de linguagem	3,600	0,507	0	0	6	9	1,000
	Pertinência prática	3,600	0,507	0	0	6	9	1,000
	Relevância teórica	3,667	0,488	0	0	5	10	1,000
**4ª visão geral - atendimento inicial (item “B”)**	Clareza de linguagem	3,733	0,458	0	0	4	11	1,000
	Pertinência prática	3,600	0,507	0	0	6	9	1,000
	Relevância teórica	3,733	0,458	0	0	4	11	1,000
**5ª atendimento inicial (itens “C”, “imagem anatômica com legenda”)**	Clareza de linguagem	3,533	0,640	0	1	5	9	0,933
	Pertinência prática	3,600	0,507	0	0	6	9	1,000
	Relevância teórica	3,667	0,488	0	0	5	10	1,000
**6ª visão geral - atendimento inicial (item “D”)**	Clareza de linguagem	3,400	0,828	0	3	3	9	0,800
	Pertinência prática	3,533	0,640	0	1	5	9	0,933
	Relevância teórica	3,600	0,632	0	1	4	10	0,933
**7ª visão geral - atendimento inicial (item “E”)**	Clareza de linguagem	3,667	0,617	0	1	3	11	0,933
	Pertinência prática	3,733	0,458	0	0	4	11	1,000
	Relevância teórica	3,733	0,458	0	0	4	11	1,000
**8ª visão geral - atendimento inicial (item “F”)**	Clareza de linguagem	3,333	0,724	0	2	6	7	0,867
	Pertinência prática	3,533	0,640	0	1	5	9	0,933
	Relevância teórica	3,600	0,632	0	1	4	10	0,933
**9ª final (itens “potenciais riscos”, “profissionais envolvidos na transição”)**	Clareza de linguagem	3,733	0,594	0	1	2	12	0,933
	Pertinência prática	3,800	0,414	0	0	3	12	1,000
	Relevância teórica	3,867	0,352	0	0	2	13	1,000

*IVC - Índice de Validade de Conteúdo; DP - desvio padrão.*

**Tabela 2:** Descrição das respostas relativas à segunda rodada de validação

Etapa	Itens	Média	DP	Respostas				IVC
				1	2	3	4	
**2ª visão geral - cinemática da ocorrência**	Clareza de linguagem	3,417	0,669	0	1	5	6	0,917
	Pertinência prática	3,500	0,674	0	1	4	7	0,917
	Relevância teórica	3,750	0,452	0	0	3	9	1,000

*IVC - Índice de Validade de Conteúdo; DP - desvio padrão.*

Mesmo com IVC adequado, algumas sugestões foram aceitas para melhorar a praticidade e segurança como: 1ª etapa: mudança do subitem “sexo” para opção de marcação, inclusão de “nome da mãe” e “número de identificação da ocorrência”; 5ª etapa: troca de símbolos na legenda para melhor distinção das lesões; 6ª etapa: descrição completa de siglas menos usadas e reorganização dos subitens para uma sequência lógica; 8ª etapa: ajuste do termo “peso” para “peso aproximado” no subitem “pediátrico”; 9ª etapa: inclusão do conselho de classe dos profissionais no item “profissionais envolvidos na transição”.

Além disso, foram feitas adequações de formatação e acréscimo de legendas ao final do instrumento.

### Descrição das respostas relativas à segunda rodada de validação

Devido ao IVC inadequado da segunda etapa (visão geral - cinemática da ocorrência), foi realizada uma nova rodada de validação, incorporando as sugestões dos especialistas: readequação do termo “cinemática” para “avaliação”, pois “cinemática” se refere ao movimento dos corpos, adequado apenas a mecanismos de trauma; inclusão de um subitem sobre o uso de dispositivos de segurança, para prever lesões não aparentes que podem ser causadas pela compressão; adição do subitem “incidentes com múltiplas vítimas” no item tipo/motivo da ocorrência, devido à complexidade e necessidade de priorizar intervenções imediatas em tais cenários; ênfase na origem e destino no subitem “transporte”, para melhor clareza e complementariedade da informação; remoção do subitem “ambiente privado”, devido a possíveis ambiguidades com “serviço de saúde”, que pode ser privado ou ocorrer em ambiente privado.

Durante a segunda rodada, 20% dos especialistas se abstiveram. A etapa revisada foi enviada em um novo formulário *online*, e os especialistas puderam novamente sugerir melhorias. A Tabela 2 mostrou um aumento no índice de consenso, com todos os itens atingindo IVC >0,9. Não houve sugestões adicionais relevantes, validando, assim, todas as etapas com sucesso pelos especialistas.

### Confiabilidade do instrumento

Na análise de confiabilidade do instrumento, mensurada por meio da avaliação do alpha de Cronbach, obteve-se o resultado de 0,93, indicando consistência interna e uma excelente confiabilidade do instrumento.

Fortalecendo a confiabilidade da avaliação realizada, foram calculados os valores do alfa de Cronbach se o item for deletado (Tabela 3), apontando-se que os valores permanecem altos, mostrando que cada item contribui expressivamente para a consistência interna do instrumento, estando fortemente correlacionados entre si e sugerindo que eles medem construtos semelhantes, contribuindo de maneira consistente para validação geral do instrumento.

**Tabela 3:** Valor do alfa de Cronbach conforme remoção do item

Etapa	Itens	Alfa de Cronbach se o item for deletado
**Identificação**	Clareza de linguagem	0,921
	Pertinência prática	0,921
	Relevância teórica	0,921
**Visão geral - cinemática da ocorrência**	Clareza de linguagem	0,922
	Pertinência prática	0,919
	Relevância teórica	0,920
**Visão geral - atendimento inicial (itens “estado geral”, “X”, “A”)**	Clareza de linguagem	0,924
	Pertinência prática	0,922
	Relevância teórica	0,922
**Visão geral - atendimento inicial (item “B”)**	Clareza de linguagem	0,922
	Pertinência prática	0,922
	Relevância teórica	0,924
**Visão geral - atendimento inicial (itens “C”, “imagem”)**	Clareza de linguagem	0,921
	Pertinência prática	0,919
	Relevância teórica	0,919
**Visão geral - atendimento inicial (item “D”)**	Clareza de linguagem	0,923
	Pertinência prática	0,922
	Relevância teórica	0,920
**Visão geral - atendimento inicial (item “E”)**	Clareza de linguagem	0,918
	Pertinência prática	0,917
	Relevância teórica	0,917
**Visão geral - atendimento inicial (item “F”)**	Clareza de linguagem	0,924
	Pertinência prática	0,919
	Relevância teórica	0,919
**Final (itens “potenciais riscos”, “profissionais envolvidos na transição”)**	Clareza de linguagem	0,924
	Pertinência prática	0,920
	Relevância teórica	0,920

## DISCUSSÃO

A complexidade da transição de tarefas impacta diretamente a carga cognitiva, influenciando a utilização de recursos psicológicos como memória, atenção, percepção, raciocínio e criatividade durante a resolução de problemas^(31)^. O uso de folhas pré-impressas tem demonstrado ser altamente benéfico para melhorar a retenção de informações durante a transição, praticamente eliminando a perda de dados^(32)^. Os *checklists* emergem como um importante recurso na prevenção de eventos adversos e promoção da segurança do paciente, contribuindo para a melhoria da comunicação, prevenção de incidentes e geração de indicadores de qualidade, especialmente em ambientes de emergência, onde o cuidado é complexo e intenso. Evidências científicas reforçam a importância da padronização de protocolos de comunicação entre profissionais de saúde^(33)^.

A revisão de literatura sobre cuidados transicionais revela preocupações intercontinentais com lacunas existentes, evidenciando a necessidade de ferramentas de comunicação padronizadas para boas práticas em saúde. Foi identificada uma preocupação com a transição de cuidados em diversos ambientes de saúde, incluindo cuidados intensivos e semi-intensivos, revelando a fragilidade deste tema em diferentes níveis de complexidade assistencial. A falta de sistematização na gestão de informações pode levar a falhas de comunicação, expondo o paciente a riscos^(34)^.

A participação ativa de toda a equipe de saúde é crucial, com a identificação de homólogos durante a transferência e a assunção de responsabilidades para garantir a continuidade do cuidado^(35)^. Mnemônicos, embora não voltados principalmente para a transição de cuidados, são úteis para a comunicação interprofissional, fornecendo estrutura para a memorização de etapas do atendimento. O SBAR, por exemplo, é amplamente utilizado e associado à melhoria da segurança do paciente^(36)^.

A construção de instrumento eficaz é fundamentada na organização e agrupamento de informações que viabilizem o pensamento crítico. Penedo^(37)^ afirma que, na prática de enfermagem, são essenciais a sistematização e o embasamento científico para uma assistência segura e direcionada. O instrumento deste estudo, com quatro categorias, como identificação, visão geral, potenciais riscos e profissionais responsáveis, visa padronizar a transferência de cuidados entre profissionais de enfermagem^(1)^.

A validação de novos instrumentos é crucial e deve ser assegurada por meio de validação de conteúdo^(21)^. No contexto brasileiro, a força de trabalho em enfermagem está rejuvenescendo, com predominância feminina, refletindo a profissão historicamente feminina^(38,39)^. A inclusão de especialistas de diferentes regiões poderia enriquecer o instrumento com diversas opiniões e sugestões^(40)^.

A variedade educacional dos participantes do estudo, com 94% possuindo pós-graduação, reflete uma riqueza de experiências e habilidades, essenciais para uma prática cientificamente fundamentada^(37,39)^. A atuação simultânea dos especialistas em assistência e docência/pesquisa assegura um julgamento teórico do conteúdo e sua aplicabilidade prática^(37)^. A concentração dos participantes em capitais destaca a influência das condições de trabalho e recursos disponíveis^(40)^.

O aprimoramento de um novo instrumento é desafiador e requer validação para assegurar que o conteúdo abranja o que se pretende mensurar. O instrumento foi estruturado para mitigar riscos de incidentes relacionados à identificação do paciente, falhas de comunicação, erros de medicação, procedimentos cirúrgicos equivocados, infecções e quedas^(8)^.

A avaliação da consistência interna, com coeficiente alfa de Cronbach de 0,93, revela excelente consistência interna, assegurando resultados válidos e confiáveis^(41)^. As sugestões dos especialistas foram essenciais para aprimorar os subitens, resultando em um conteúdo pertinente e relevante, validado com sucesso e alinhado com as evidências científicas.

### Limitações do estudo

As limitações deste estudo incluem a subjetividade da análise, uma vez que as avaliações dos especialistas podem ser influenciadas por opiniões pessoais, resultando em distorções nos dados de validação. Além disso, o número limitado de especialistas (15 e 12) pode não representar adequadamente a diversidade de experiências, afetando a generalização dos resultados. A validação de conteúdo aborda apenas aspectos de clareza, pertinência e relevância, sem incluir outras medidas psicométricas que poderiam fornecer uma avaliação mais abrangente da eficácia. Restrições de tempo e recursos podem ter limitado o desenvolvimento e a validação da tecnologia, impactando a profundidade do estudo. Por fim, a implementação da tecnologia na prática clínica pode enfrentar desafios, como resistência à mudança por parte dos profissionais ou falta de treinamento adequado.

### Contribuições para as áreas da saúde, enfermagem ou políticas públicas

Ao fornecer uma tecnologia assistiva que sincroniza a comunicação entre os enfermeiros durante a transição do cuidado, este estudo contribuirá com a enfermagem e reduzirá grandes erros e ineficiências na sala de emergência. A tecnologia de segurança do paciente melhora a qualidade e a eficiência dos cuidados, fornecendo informações claras e precisas para reduzir erros, e promovendo uma cultura de segurança. Além disso, como ferramenta educacional, capacita enfermeiros nas melhores práticas de comunicação e colaboração profissional e na gestão da integração dos serviços de saúde. Como ferramenta de aplicação nacional, pode ser utilizada em diversos contextos e promover consistência e eficácia.

## CONSIDERAÇÕES FINAIS

A prevenção de falhas e omissões na emergência requer uma compreensão profunda das suas origens, que podem incluir falhas de raciocínio, deslizes e lapsos, exacerbadas por fatores como fadiga, ritmo acelerado, horas extras, sobrecarga de trabalho e alta rotatividade de funcionários. Identificando uma lacuna na segurança do paciente durante a transição de cuidados entre serviços pré-hospitalares móveis e portas de urgência, julgou-se relevante desenvolver e validar uma tecnologia assistencial que abordasse de forma crítica os principais conteúdos durante a prestação de assistência e garantisse a continuidade do cuidado em todos os níveis.

A “Tecnologia Assistencial para a Comunicação Efetiva durante a Transição de Cuidados” foi criada como uma solução para melhorar a qualidade, efetividade e segurança do atendimento. Esta tecnologia se destaca como uma estratégia de baixo custo e fácil aplicação, promovendo uma cultura de segurança do paciente e fortalecendo as relações interprofissionais.

O processo de validação revelou que houve consenso entre os especialistas, com IVCs superiores a 0,90, em sua maioria, refletindo sua clareza, pertinência prática e relevância teórica. Ademais, a análise de confiabilidade do instrumento, por meio do coeficiente alfa de Cronbach, demonstrou um valor de 0,93, evidenciando excelente consistência interna.

Os resultados desta pesquisa mostram que a tecnologia possui validade de conteúdo em relação à clareza de linguagem, pertinência prática e relevância teórica, destacando sua importância. Esta tecnologia se apresenta como um diferencial por oferecer uma estratégia padronizada de comunicação interprofissional, sendo um valioso instrumento de aplicação nacional que busca estabelecer um processo comunicativo seguro.

Este instrumento marca um passo importante na busca por uma assistência sistematizada que assegure a continuidade integral dos cuidados em saúde. Em etapas subsequentes, o instrumento será submetido a novos testes para avaliar sua eficácia quando aplicado por profissionais emergencistas.

Contudo, é importante reconhecer que todo estudo de validação de conteúdo pode ter limitações devido à natureza subjetiva da análise dos especialistas, o que pode resultar em distorções. Portanto, embora a validade de conteúdo ofereça informações sobre a representatividade e clareza de cada item com base na colaboração dos especialistas, ainda é necessário aplicar outras medidas psicométricas adicionais.

Assim, para viabilizar a submissão a outras etapas de validação e futura implementação na rotina dos profissionais de emergência, recomenda-se a realização de novos estudos para buscar mais evidências da aplicabilidade do instrumento.

## Data Availability

Não se aplica.
